# Metal-Organic Framework-Based Materials in Aqueous Zinc-Ion Batteries

**DOI:** 10.3390/ijms24076041

**Published:** 2023-03-23

**Authors:** Fuhai Wu, Buke Wu, Yongbiao Mu, Binbin Zhou, Guobin Zhang, Lin Zeng

**Affiliations:** 1 Shenzhen Key Laboratory of Advanced Energy Storage, Southern University of Science and Technology, Shenzhen 518055, China; 2 Department of Mechanical and Energy Engineering, Southern University of Science and Technology, Shenzhen 518055, China; 3 SUSTech Energy Institute for Carbon Neutrality, Southern University of Science and Technology, Shenzhen 518055, China; 4 Shenzhen Institute of Advanced Electronic Materials, Shenzhen Institute of Advanced Technology, Chinese Academy of Sciences, Shenzhen 518055, China; 5 Tsinghua Shenzhen International Graduate School, Tsinghua University, Shenzhen 518055, China; whutzgb@163.com

**Keywords:** metal-organic frameworks, MOF derivatives, aqueous zinc-ion batteries, cathode, anode

## Abstract

Aqueous zinc-ion batteries (AZIBs) are promising for large-scale energy storage systems due to their high safety, large capacity, cost-effectiveness, and environmental friendliness. However, their commercialization is currently hindered by several challenging issues, including cathode degradation and zinc dendrite growth. Recently, metal-organic frameworks (MOFs) and their derivatives have gained significant attention and are widely used in AZIBs due to their highly porous structures, large specific surface area, and ability to design frameworks for Zn^2+^ shuttle. Based on preceding contributions, this review aims to generalize two design principles for MOF-based materials in AZIBs: cathode preparation and anode protection. For cathode preparation, we mainly introduce novel MOF-based electrode materials such as pure MOFs, porous carbon materials, metal oxides, and their compounds, focusing on the analysis of the specific capacity of AZIBs. For anode protection, we systematically analyze MOF-based materials used as 3D Zn architecture, solid electrolyte interfaces, novel separators, and solid-state electrolytes, highlighting the improvement in the cyclic stability of Zn anodes. Finally, we propose the future development of MOF-based materials in AZIBs. Our work can give some clues for raising the practical application level of aqueous ZIBs.

## 1. Introduction

Owing to a sizable amount of fossil fuel consumption, the world faces the challenge of an energy crisis and environmental deterioration. Subsequently, renewable energies such as wind, solar, and wave energy have been investigated extensively in recent years [1]. In the meantime, developing energy storage systems (ESSs) cannot only overcome the non-continuity shortcoming of the abovementioned energy resources but also enable stable energy supplies [2]. Among various ESSs, electrochemical energy storage (EES) technologies, including lithium-ion batteries, supercapacitors, and zinc-ion batteries, play a promising role in the energy revolution [3]. Especially, lithium-ion batteries (LIBs) have achieved great success in commercialization and have occupied the dominant position in portable electronic devices and electric vehicle markets due to their high energy density and long service life [4,5]. However, their future large-scale applications are still impeded by some significant drawbacks [6,7,8]: (1) high prices owing to the expensive electrode materials and stringent cell assembly conditions; (2) safety hazards from highly toxic and flammable organic electrolytes; (3) limited resources of the raw lithium and cobalt materials in nature. Considering the above bottlenecks, LIBs are not available as energy storage systems at the grid level [9]. Therefore, researchers focus on developing aqueous mental/non-mental ion batteries (Na^+^ [10], K^+^ [11], Zn^2+^ [12], Mg^2+^ [13], H^+^ [14], Cl^−^ [15], etc.) due to their higher safety and lower cost in meeting the ever-growing demands of the energy industry.

**Figure 1:**
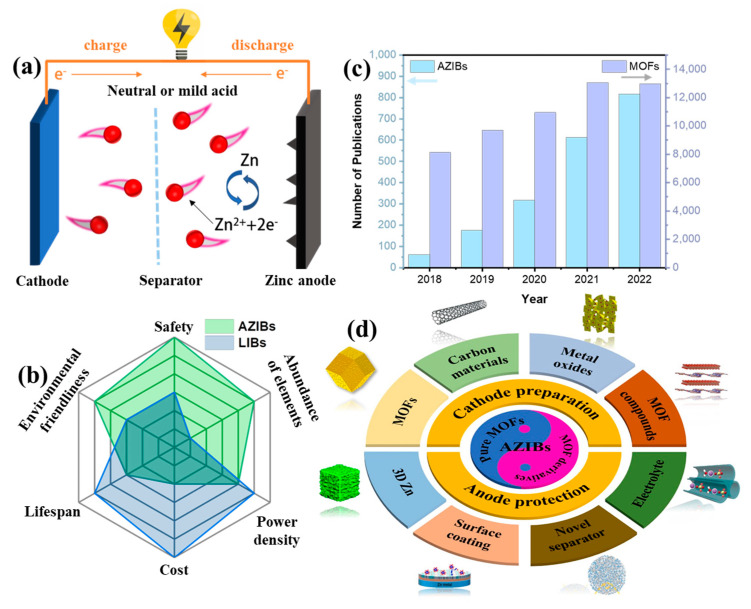
(**a**) Mechanism of AZIBs. (**b**) Multi-angle comparison of AZIBs and LIBs. (**c**) Total number of scientific publications on MOFs and AZIBs published in recent years (data collected form Clarivate Web of Science; keywords used: “metal-organic frameworks”, “aqueous zinc-ion batteries”). (**d**) Graphical abstract of this work.

Fortunately, among the various aqueous batteries mentioned above, aqueous zinc-ion batteries (AZIBs) hold great promise as the candidate for large-scale energy storage applications featuring several benefits [16,17,18,19,20]: (1) cost-effectiveness; Zn metal is inexpensive due to its natural abundance, and an open-air manufacturing environment lowers the production cost; (2) intrinsic safety; neutral or mildly acidic aqueous electrolytes are innocuous and nonflammable; (3) zinc metal’s proper redox potential (−0.763 V vs. standard hydrogen electrode); Zn metal shows higher stability and excellent Zn/Zn^2+^ reversibility in aqueous media due to its appropriate redox potential; (4) high capacity: Zn metal has a high theoretical capacity (820 mAh g^−1^ and 5854 mAh cm^−3^). Importantly, compared to other aqueous mental/non-mental ion batteries and different EES technologies, AZIBs possess a relatively higher energy–power density combination [21,22], which indicates that the development of AZIBs is of great promise. As shown in Figure 1a, AZIBs have a similar charge and discharge mechanism as LIBs: Zn^2+^ acts as a carrier in the circuit, reversibly extracting/inserting (cathode) and plating/stripping (anode) upon charging/discharging [23,24,25,26]. Differently, AZIBs have more potential prospects for large-scale applications due to multi-angle superiorities over LIBs (Figure 1b). Currently, the practical applications of AZIBs still face enormous challenges, mainly including the following factors [27,28,29]: (1) the rapid capacity decay is accompanied by the cathode degradation during the repeated interaction and extraction of Zn^2+^/H^+^ ions; (2) the undesirable dendrite formation, corrosion, and hydrogen evolution reaction (HER) on the Zn anode is irreversible, which hinders the cyclability of the zinc anode; (3) the operational voltage of AZIBs is restricted by narrow electrochemical stability window of aqueous electrolytes. To solve these issues, scholars have conducted numerous research activities on designing rational functional materials to enhance the specific capacity and cyclic stability of AZIBs [30].

As a new type of molecular crystal materials, metal-organic frameworks (MOFs) have already attracted much attention, as evidenced by the growth of related publications in the last 5 years (Figure 1c). MOFs are a kind of coordination polymer that have a three-dimensional pore structure by self-assembling metal ions as the connecting points and organic ligands [31,32]. From the first invention by Yaghi and coworkers in 1995 [33] to now, pure MOFs and their derivatives have made up the abundant family of MOF-based materials [34,35,36]. For instance, novel porous carbon materials can be architected by using mother MOFs as templates or precursors [37,38]. It is worth noting that MOF-based materials are suitable in the application of AZIBs for several advantages [39,40,41]: (1) a high porosity enables stability by reducing volume change and is beneficial for electrolyte penetration; (2) a large specific surface area can provide abundant redox active sites to maximize the interphase between electrode and electrolyte and promote reaction kinetics; (3) the character of tailor can achieve adjustable functionality as needed, for example, the pore size can be adjusted by changing the length of organic ligands, which helps to mass transfer; (4) coordination diversity avails to select proper electrode materials by changing the components of metal centers and organic linkers; (5) structural topologies provide decent charge/discharge capability and cycle life, for example, a hollow structure can alleviate the effects of the pulverization of active material. Encouraged by these advantages, there has been a vast expansion of publications that showcase the latest progress in employing MOF-based materials for improving the specific capacity and cyclic stability of AZIBs. For example, Wang et al. reported a 2D sandwich-like MOF/MXene composite for durable and fast aqueous zinc-ion batteries [42]. After carefully scrutinizing previous reports, herein we generalize two principles about the design of MOF-based materials for promoting AZIBs, namely cathode preparation and anode protection (Figure 1c). For cathode preparation, multifarious advanced MOF-based cathode materials (pure MOFs, porous carbon materials, metal oxides, and others) for high-performance AZIBs are comprehensively summarized in this contribution, focusing on the analysis of the specific capacity. Moreover, we review the approaches for anode protection with MOF-based materials in terms of constructing 3D zinc architecture, solid electrolyte interfaces (SEI), novel separators, and solid-state electrolytes (SSE), highlighting the improvement in the cyclic stability of Zn anodes. Finally, the development trend of how MOF-based materials could better complement AZIBs is put forward. This review plays an important role in the development of MOF-based materials in the field of AZIBs.

## 2. MOF-Based Materials for Cathode Preparation in AZIBs

Currently, the research on MOF-based materials has become a hot topic due to their versatile compositions, high surface areas, tunable structures, uniform dispersion, and defined active centers [43]. More and more scientists have employed MOF-based materials as alternative electrodes [44,45,46]. In this section, we summarize the advanced progress of MOF-based materials for AZIBs cathodes from four aspects: pure MOFs, carbon materials, metal oxides, and MOF compounds.

### 2.1. Pure MOFs for Cathode Preparation

**Figure 2:**
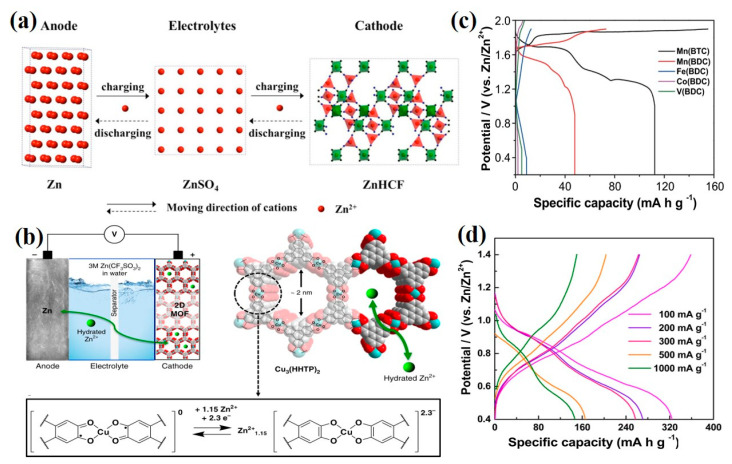
Schemes follow the same formatting. (**a**) A schematic of the Zn//ZnHCF battery [49]. Copyright 2014, John Wiley and Sons. (**b**) Structure and expected redox process in the coordination unit of Cu_3_(HHTP)_2_ [50]. Copyright 2019, Springer Nature. (**c**) Charge/discharge curves at 50 mA g^−1^ of Mn(BTC), Mn(BDC), Fe(BDC), Co(BDC), and V(BDC) cathodes [51]. Copyright 2020, Springer Nature. (**d**) Galvanostatic charge/discharge profile of V-MOF (MIL-47) at different current densities [52]. Copyright 2021, Elsevier.

MOFs with high specific surface area and low density are promising electrode materials for AZIBs [47]. For instance, Prussian blue analogues (PBAs) are a type of coordination compound with a 3D open-framework structure, which can be described as MHCF (M are transition metal ions, and HCF means the hexacyanoferrate) [19]. According to the definition of MOFs, it is reasonable to classify PBAs as MOFs [48]. Zhang et al. synthesized rhombohedral zinc hexacyanoferrate (ZnHCF) and investigated its electrochemical properties as the cathode in AZIBs for the first time (Figure 2a) [49]. When the ZnHCF cathode was combined with a zinc anode, the full battery average operating voltage was as high as 1.7 V. Galvanostatic measurements displayed that the ZnHCF cathode can deliver a capacity of 65.4 mAh g^−1^ at 60 mA g^−1^ and good stability with a capacity retention of over 81% at 300 mA g^−1^. Through XPS and ex situ XRD techniques, the intercalation of Zn^2+^ ions into ZnHCF was verified. These key results pave the way for the further exploration of MOF-based cathodes in AZIBs. Moreover, a conductive two-dimensional MOF Cu_3_(HHTP)_2_ (HHTP is 2,3,6,7,10,11-hexahydroxytriphenylene) was designed by Nam and coworkers (Figure 2b) [50]. The electrical conductivity (0.2 S cm^−1^) and large one-dimensional channels (pores of ~2 nm) that existed in Cu_3_(HHTP)_2_ can fasten the diffusion of electron and Zn^2+^ ion to active sites and reduce interfacial impedance. The charge/discharge curves demonstrated that the Cu_3_(HHTP)_2_ cathode displayed a high reversible capacity of up to 228 mAh g^−1^ at 50 mA g^−1^ and outstanding capacity retention of 75% after 500 cycles at a high current density of 4 A g^−1^. Furthermore, the capacitive currents contribution of Cu_3_(HHTP)_2_ obtained by CV measurements was 83%, indicating that the Cu_3_(HHTP)_2_ follows an intercalation pseudocapacitance charge storage mechanism. In addition, Pu et al. synthesized five various MOF materials (denoted as Mn(BTC), Mn(BDC), Fe(BDC), Co(BDC), and V(BDC)) and systematically evaluated their electrochemical behaviors as the cathodes of AZIBs [51]. The charge/discharge curves revealed that Mn(BTC) exhibited the highest Zn^2+^ storage capacity of 112 mAh g^−1^ at a current of 50 mA g^−1^ (Figure 2c). Characterized by XRD, SEM, XPS, and FTIR, the transformation from Mn(BTC) to Zn(BTC) was observed during the first charge process while the Mn^2+^ ions dissolved into aqueous electrolytes and were oxidized to MnO_2_ on the cathode surface, serving as a host for Zn^2+^ and H^+^ storage in the following charge/discharge processes. Interestingly, rod-like Zn(BTC) was beneficial to the ion diffusion and cycle life. By adding MnSO_4_ to the ZnSO_4_ electrolyte, the resultant battery showed a long cycle life with 92% capacity retention after 900 cycles at 1000 mA g^−1^. Moreover, a particular type of V-MOF (MIL-47, V^IV^(O)(bdc)) featuring a one-dimensional layered nanorod-like framework was fabricated by Ru et al. via a facile one-pot hydrothermal method [52]. The as-prepared V-MOF was equipped with an enormous number of empty channels, which enabled it to increase the reaction active sites and boosted Zn^2+^ insertion and extraction. The assembled Zn//V-MOF battery delivered a specific discharge capacity of 332.3 mAh g^−1^ at 0.1 A g^−1^ (Figure 2d). Obviously, assorted pure MOFs as cathode materials show discrepant specific capacity by matching different metal clusters and organic ligands, the appropriate pairing needs to be further explored.

**Figure 3:**
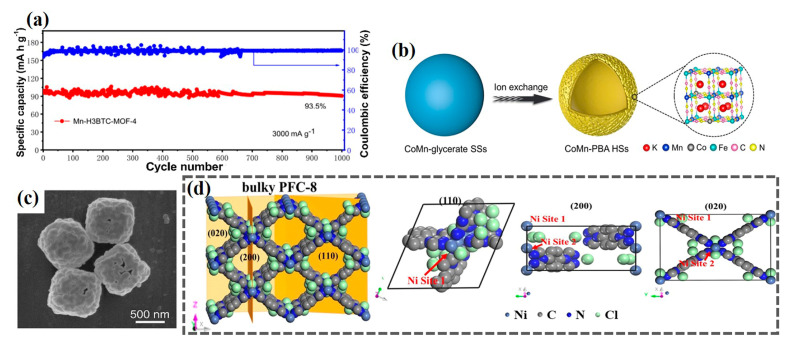
(**a**) Cycling performance of Mn-H3BTC-MOF-4 as the cathode for AZIBs at 3000 mA g^−1^ [53]. Copyright 2021, American Chemical Society. (**b**) The synthetic process of CoMn-PBA HSs [54]. Copyright 2021, John Wiley and Sons. (**c**) SEM image of CuMn-PBA DSNBs [55]. Copyright 2021, John Wiley and Sons. (**d**) Atomic structure of the PFC-8 framework with an illustration of (110)-, (200)-, and (020)-exposed crystal facets [56]. Copyright 2022, John Wiley and Sons.

Additionally, it is worth mentioning that there are some optimization designs on the inner structure of pure MOFs to improve their electrochemical activity. Succeeding the research of Pu and coworkers mentioned above, Yin et al. innovatively demonstrated the idea of coordinately unsaturated Mn(BTC) as the cathode candidate of AZIBs [53]. By adjusting the Mn and −COOH with the molar ratio of 1.32:4, the optimal Mn-H3BTC-MOF-4 contributed to efficient Zn^2+^ transport and electronic/ionic conductivity. As a result, it showed a high capacity of 138 mAh g^−1^ at 0.1 A g^−1^ and 6.5% capacity fading after 1000 cycles at 3 A g^−1^ (Figure 3a). Moreover, Zeng and coworkers rationally designed Co-substituted Mn-rich PBA hollow spheres (denoted as CoMn-PBA HSs) [54] and Cu-substituted Mn-PBA double-shelled nanoboxes (denoted as CuMn-PBA DSNBs) [55] through an ion exchange approach (Figure 3b,c). On the one hand, the unique hollow structure of materials exposed rich active sites, which alleviated the volume change during cycle performance. On the other hand, partial metal ions substitution might inhibit the Jahn–Teller distortions of Mn-N_6_ octahedra, thus contributing to the prolonged lifespan. The as-prepared CoMn-PBA HSs cathode exhibited a high reversible capacity of 128.6 mAh g^−1^ at 50 mA g^−1^. Similarly, the CuMn-PBA DSNBs cathode delivered a capacity of 116.8 mAh g^−1^ at 0.1 A g^−1^. Moreover, the exposed facet regulation of Ni-based MOF (PFC-8) was proposed by Yang et al. through a thermally modified strategy [56]. Generally, the PFC-8 was dominated by the exposed (110) facet [57]. After being heated to 350 °C and cooled down to room temperature, XRD characterization identified that (200) and (020) facets of PFC-8 350 increased significantly, which was favorable for specific capacity and electrochemical kinetics on account of (200) and (020) facets having double the Ni sites (acting as the active sites) than (110) facets (Figure 3d). Consequently, the PFC-8 350 cathode achieved a superior discharge capacity of 110.0 mAh g^−1^ at 30 A g^−1^ while the PFC-8 cathode was 29.7 mAh g^−1^. As discussed above, pure MOFs with fascinating morphology and pore characteristics dramatically improve the electrochemical performance of AZIBs. Nevertheless, several disadvantages curtail the future development of pure MOFs, such as poor electric conductivity and structural instability. The poor electrical conductivity is an intrinsic consequence of how MOFs are typically constructed and structural instability results from their collapse under harsh charge/discharge conditions. To tackle these issues, MOF derivatives have been extensively considered as cathode materials for AZIBs, such as porous carbon materials, metal oxides, and their compounds.

### 2.2. MOF-Derived Carbon Materials for Cathode Preparation

**Figure 4:**
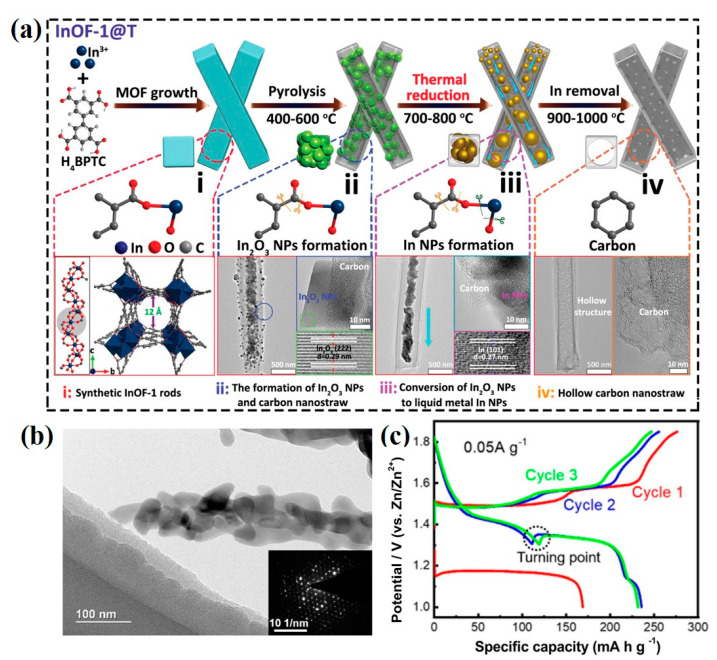
(**a**) Schematic illustration to obtain InOF-1-derived hierarchically porous HCNS with their differentiated carbonized stages at different temperatures: (**i**) Synthetic InOF-1 rods; (**ii**) The formation of In_2_O_3_ NPs and carbon nanostraw; (**iii**) Conversion of In_2_O_3_ NPs to liquid metal In NPs; (**iv**) Hollow carbon nanostraw [65]. Copyright 2022, John Wiley and Sons. (**b**) TEM image of as-prepared α-Mn_2_O_3_ (the inset is electron diffraction pattern). (**c**) Capacity-voltage profiles of α-Mn_2_O_3_ at 0.05 A g^−1^ [69]. Copyright 2021, Elsevier.

Carbon materials such as carbon nanotubes (CNTs) [58,59], carbon fiber [60,61], and graphite/graphene [62,63] can effectively improve the electrochemical performance of electrode active materials. Among them, CNTs have a dense tube wall structure and slender tube diameter structure, which largely restrict the effective diffusion and electron transmission of internal active materials and electrolyte ions [64]. Therefore, Chai et al. in situ obtained a type of hierarchically porous hollow carbon nanostraw (denoted as HCNS) via facile pyrolysis and thermal reduction in an indium-based organic framework InOF-1 [65]. The synthesis illustration of HCNS is given in Figure 4a. Compared with traditional CNTs, the MOF-derived HCNS stored more charge active sites and shortened ion transport pathways, which was favorable for better electrochemical exchange capacity. As a consequence, the zinc–iodine batteries assembled with as-synthesized HCNS displayed a maximum discharge capacity of 295.7 mAh g^−1^ at 0.5 A g^−1^ and a high Coulombic efficiency (87% after 1500 cycles) at 1 A g^−1^. In addition, carbon materials are widely used in MOF compounds such as MnO/C@rGO [66], MnO_2_/CC [67], and V_2_O_5_@C [68], which is beneficial to enhance the electrical conductivity of composite cathode materials. However, these pristine carbon materials only provide limited physical trappings of active materials. Instead, MOF-derived carbon materials with suitable size and exceptionally large surface areas provide a great possibility to mitigate the shuttle effect by chemically interacting active materials with higher binding energies, which contribute to the superior electrochemical performance of electrodes.

### 2.3. MOF-Derived Metal Oxides for Cathode Preparation

Traditional metal oxides, such as V_2_O_5_, and MnO_2_, have been extensively investigated as cathodes for AZIBs because of their high theoretical capacity. However, severe structural degradation of these materials limited their zinc storage capacity and rate capability. MOF-derived metal oxides with large specific surface areas and sufficient electrochemical active sites are feasible cathode materials. For instance, α-Mn_2_O_3_ was explored as a cathode of AZIBs by Mao and coworkers through the Mn-BTC-derived method [69]. TEM image showed that α-Mn_2_O_3_ exhibited rod-like morphology, consisting of nanoparticles with a diameter of about 100 nm (Figure 4b), which boosted the contact between the cathode and electrolyte for fast ion diffusion. When assembled as a Zn/α-Mn_2_O_3_ battery, it obtained a high specific capacity of 225 mAh g^−1^ at 0.05 A g^−1^ (Figure 4c). Impressively, this work pointed out the relationship between the charge storage mechanism of the α-Mn_2_O_3_ cathode and discharge current density. At lower current densities, the H^+^ and Zn^2+^ were intercalated cooperatively, while the H^+^ intercalation occurred dominantly at higher current densities. Additionally, MOF-derived V_2_O_3_ [70], ZnMn_2_O_4_/Mn_2_O_3_ [71], and Mn_2_O_3_/Al_2_O_3_ [72] were developed as well, and all of them enhanced the capacity and cycling stability of AZIBs successfully. Apparently, nanostructured MOF-derived metal oxides demonstrate significantly improved H^+^/Zn^2+^ storage performance as cathode materials compared with that of traditional metal oxides due to their unique structures, which provide abundant active sites and is favorable for excellent high capacity.

### 2.4. MOF Compounds for Cathode Preparation

**Figure 5:**
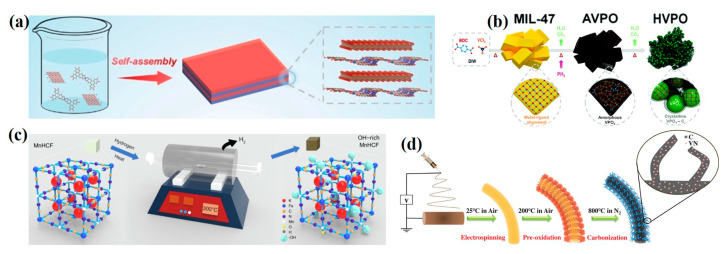
Schematic illustration of the formation process of (**a**) Cu-HHTP/MX [42]. Copyright 2023, John Wiley and Sons. (**b**) HVPO [74]. Copyright 2022, Royal Society of Chemistry. (**c**) OH-rich MnHCF [73]. Copyright 2023, Elsevier, and (**d**) VN/N-CNFs [75]. Copyright 2022, John Wiley and Sons.

MOF compounds can concoct pure MOFs, metal oxides, carbon materials, or other functional materials/groups together. This hybrid strategy can engender synergistic effects and optimize electrode performance to the maximum extent. For instance, a sandwich-like and alternately stacked Cu-HHTP and MXene heterostructure was designed by Wang et al. (Figure 5a) [42]. The Cu-HHTP/MX composite inherited advantages of both MOFs and MXene, which not only provided abundant active sites but also enhanced the electrical conductivity for effective charge transport. DFT calculations unraveled the highly reversible Zn^2+^ storage and zero-strain feature of the Cu-HHTP/MX heterostructure as the cathode for AZIBs. Significantly, the Cu-HHTP/MX cathode realized a high reversible specific capacity of 260.1 mA h g^−1^ at 0.1 A g^−1^. In addition, Tan et al. proposed a novel hydroxylation strategy for PBA manganese hexacyanoferrate (MnHCF) (Figure 5c) [73]. During the annealing engineering under the H_2_ atmosphere, an abundance of -OH functional groups preferred to settle on Mn atoms thanks to the lowest adsorption energy (about −4.14 eV) for -OH in Mn sites. Thus, OH-rich MnHCF can stimulate the Mn^3+^/Mn^2+^ and Fe^3+^/Fe^2+^ redox reaction, thereby enhancing electrochemical kinetics. Remarkably, an impressive discharge capacity of 136.1 mAh g^−1^ and a considerable energy density of 228.8 Wh kg^−1^ at 100 mA g^−1^ was achieved. Moreover, VPO_4_ was investigated as a cathodic material for AZIBs for the first time by Hwang et al. [74]. By carbonizing and phosphating MIL-47, high-crystallinity vanadium phosphate (denoted as HVPO) nanoparticles were in situ formatted (Figure 5b). The electrically conductive carbon network not only contributed to the uniform interconnection of HVPO nanoparticles but also boosted charge transfer kinetics. During the electrochemical performance test, the HVPO cathode displayed a superior rate capability and ultra-stable capacity retention (almost no capacity fading at 10 A g^−1^ for 20,000 cycles). Moreover, Zhang et al. designed vanadium nitride-embedded nitrogen-doped carbon nanofiber (VN/N-CNFs) composites via an electrospinning technique (Figure 5d) [75]. Vanadium nitride (VN) can achieve a maximum of two-electron redox for vanadium atoms and a high theoretical specific capacity of up to 825 mAh g^−1^. By introducing MIL-47 as precursors, VN nanograins in situ grew and were homogeneously distributed into electrospun carbon nanofibers (CNFs). 3D self-supported skeletons and hierarchical structures were realized by this design strategy, which rendered a conductive layer and prevented VN nanograins from self-aggregation. Excitingly, the reversible capacity of VN/N-CNFs composites reached 734 mAh g^−1^ at 0.5 A g^−1^ and 482 mAh g^−1^ at 50 A g^−1^, and 297 mAh g^−1^ at a high rate of 100 A g^−1^. As described above, manifold MOF compounds were developed via matching assorted substances. Basically, MOF compounds as cathodes for AZIBs perform much better electrochemical behavior than their single components, shedding light upon an effective way toward superior aqueous zinc-ion batteries.

**Figure 6:**
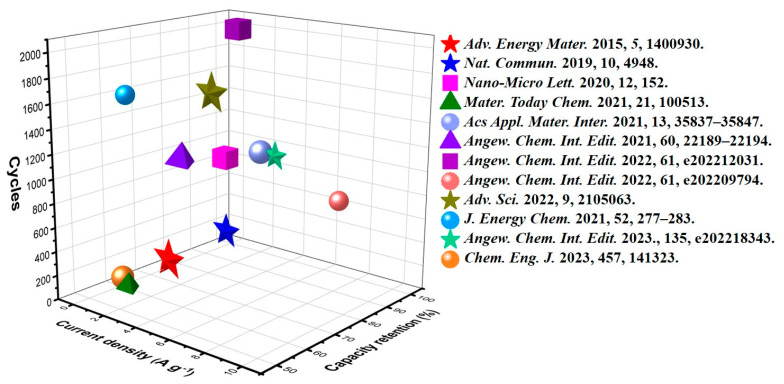
Comparison of cycle stability obtained in works [42,49,50,51,52,53,54,55,56,65,69,73] reviewed above.

In brief, we have reviewed the recent progress of MOF-based materials for cathode preparation in AZIBs (Figure 6). Due to their promising features, MOF-based cathode materials facilitate electronic and ionic transportation through the charging and discharging cycles of aqueous zinc-ion batteries, which can provide enlightenment in optimizing the reaction mechanism and specific capacity.

## 3. MOF-Based Materials for Anode Protection in AZIBs

Recently, MOF-based materials have been extensively applied for addressing the issues of zinc anodes (dendrite growth, corrosion, and hydrogen evolution, etc.). The mainstream strategies include the construction of 3D Zn architecture, surface coating, novel separators, and electrolyte engineering. These advanced strategies can effectively regulate the reversible Zn^2+^ plating and stripping process. In the next sections, we will describe the practical application of MOF-based materials for anode protection in AZIBs at length.

### 3.1. MOF-Based Materials in Constructing 3D Zn Architecture

**Figure 7:**
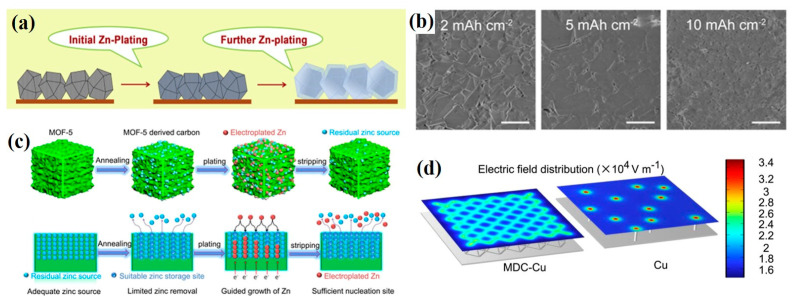
(**a**) Schematic illustration of the Zn plating on ZIF-8-500 electrode. (**b**) SEM images of Zn deposits at 1.0 mA cm^−2^ for different capacities [76]. Copyright 2019, Elsevier. (**c**) Schematic diagram of the electroplating/stripping principle of MDC. (**d**) Simulation of the electric field distribution of MDC–Cu and a planar bare Cu foil [77]. Copyright 2022, John Wiley and Sons.

It is well known that MOFs possess three-dimensional network structures, which significantly facilitate the construction of 3D Zn architecture, especially Zn-MOFs. By reducing the Zn^2+^ nodes inside MOFs into Zn^0^, an advanced Zn anode with 3D architecture is obtained. In contrast with the conventional etching strategy, the MOF-reduction strategy is accurate and cost effective. For instance, Wang and coworkers annealed ZIF-8, consisting of Zn^2+^ ions and 2-methylimidazolate, at an optimized temperature of 500 °C (denoted as ZIF-8-500) [76]. After the heat treatment, Zn^0^ reduced from Zn^2+^ was uniformly distributed in the framework of ZIF-8 without any changes to the inherent porous structure. The ZIF-8-500 anode presented ultra-stable Coulombic efficiencies (CE) up to 99.8% over 200 cycles regardless of current densities because it can serve as the substrate after the initial plating, which leads to uniform Zn nucleation for the further plating process, as illustrated in Figure 7a, and confirmed by SEM images of Zn deposits (Figure 7b). Apart from that, MOF-5 was reduced and simultaneously carbonized by Li et al. [77]. The novel anode (denoted as MDC) featuring sufficient nucleation sites can guide the zinc growth (Figure 7c) and maintain high Zn^0^ plating/stripping reversibility with an average CE of 99.4% after 3000 cycles at 1 mA cm^−2^ and 0.5 mAh cm^−2^. The finite element simulation was performed to further explain the relatively more uniform electric field and local current density distribution of the MDC anode (Figure 7d). When coupled with a Zn-containing cathode (Zn/Mn-MOF@CNT), the full cell possessed a high specific discharge capacity of 459.9 mA h g^−1^. Evidently, the construction of 3D Zn architecture with MOF-based materials is beneficial to Zn plating/stripping and the cyclic stability of AZIBs because 3D porous structured electrode can simultaneously decrease several major resistances including ion and electron transport between electrolyte and electrode and electrochemical reactions at the electrode. Therefore, it is a feasible route to enhance the electrochemical performance of AZIBs by constructing 3D Zn architecture with MOF-based materials.

### 3.2. MOF-Based Materials in Surface Coating

**Figure 8:**
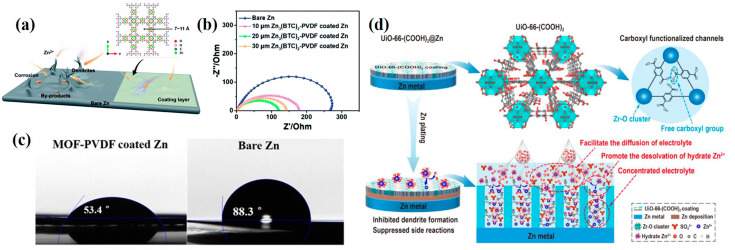
(**a**) Schematic illustration of the morphology evolution of bare and Zn-BTC coated Zn anodes during Zn stripping/plating and (**b**) EIS spectra of the symmetric cells [78]. Copyright 2022, Royal Society of Chemistry. (**c**) Images of contact angles between the electrolyte and different anodes [79]. Copyright 2019, American Chemical Society. (**d**) Schematic illustration of the functional mechanism of the UiO-66-(COOH)_2_ coating layer for protecting the Zn anode [80]. Copyright 2023, Elsevier.

Surface coating aims to form a solid electrolyte interface (SEI) between the Zn metal anode and electrolyte. However, the interface contact cannot be completely blocked, for the interphase is exactly the location where the electrochemical reactions take place. Porous MOF-based materials are appropriate candidates with blocking and dredging dual functions. Wang et al. proposed zinc benzene tricarboxylate (Zn-BTC) for Zn anode coating via a simple doctor blade method with PVDF binder (Figure 8a) [78]. After pairing with the Zn-BTC protective layer, the Zn^2+^ cations transport was accelerated while the entrance of the electrolyte anions was obstructed. Figure 8b shows that the interfacial charge transfer impedance R_ct_ value of the Zn@Zn-BTC anode was much lower than bare Zn. Subsequently, the Zn@Zn-BTC symmetric cell exhibited a superior lifespan of 800 h, and the MnO_2_//Zn@Zn-BTC full battery obtained a high specific capacity of 220 mA h g^−1^. In addition, hydrophilic microporous UiO-66 (Zr_6_O_4_(OH)_4_(BDC)_6_) MOF nanoparticles were developed to reconstruct the Zn/electrolyte interface by Liu and coworkers [79]. The addition of UiO-66 significantly lowered the wetting angle of the anode to 53.4° by contrast with bare Zn (88.3°, Figure 8c), and the R_ct_ value was reduced from 2500 to 530 Ω. Voltage curves of the Zn plating process revealed that the Zn nucleation overpotential on bare Zn is 74 mV at 2 mA cm^–2^, much higher than 29 mV with the presence of the UiO-66 coating layer. Based on UiO-66 coating, Xin et al. functionalized UiO-66 MOF with carboxyl groups, which can assist the desolvation of hydrated Zn^2+^-(H_2_O)_6_, as an ion-conductive interphase to stabilize the Zn anode (Figure 8d) [80]. The ionic conductivity of UiO-66-(COOH)_2_ was measured to be 1.91 mS cm^−1^, higher than that of UiO-66 (0.23 mS cm^−1^) by a factor of eight. When it came to the Zn^2+^ transfer number, the value for UiO-66 of 0.13 was lower than that for UiO-66-(COOH)_2_ of 0.55 as well, supporting that the UiO-66-(COOH)_2_ layer can facilitate the transfer rate of Zn^2+^ ions. Impressively, the Zn@UiO-66-(COOH)_2_ anode showed a lifespan of over 2800 h at 2 mA cm^−2^ within 2 mAh cm^−2^ while the bare Zn and Zn@UiO-66 anodes only worked for 114 h and 810 h, respectively. As is shown above, surface coating with MOF-based materials on Zn foil can observably regulate the contact between electrolyte and electrode, which is beneficial to achieve stable Zn deposition and suppress the emergence of adverse reactions, simultaneously enhancing battery performance.

### 3.3. MOF-Based Materials in Designing Novel Separators

**Figure 9:**
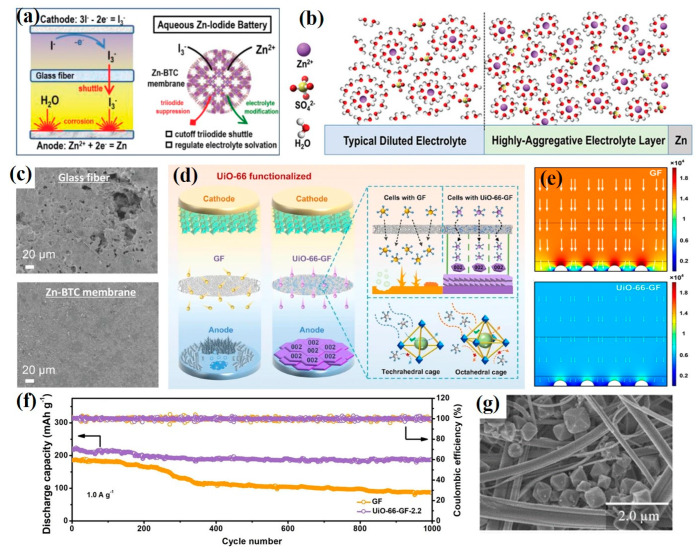
(**a**) Schematic illustration of Zn–I_2_ batteries with glass fiber (GF) separator and Zn-BTC membrane. (**b**) Solvation structure of typical diluted ZnSO_4_ solutions and high-aggregative electrolyte layer on Zn anode. (**c**) SEM images of Zn anode after cycling with GF separator and Zn-BTC membrane [81]. Copyright 2020, John Wiley and Sons. (**d**) Schematic illustration of AZIBs with UiO-66 functionalized glass fiber separator. (**e**) The electrical field models based on GF and UiO-66-GF separators. (**f**) Cycling performances and CEs of Zn|GF|MnO_2_ and Zn|UiO-66-GF|MnO_2_ cells at 1.0 A g^−1^ [82]. Copyright 2022, Springer Nature. (**g**) SEM images of UiO-66 modified a glass microfiber separator [83]. Copyright 2022, Elsevier.

The separator is of great importance to AZIB performances since it can prevent contact between two electrodes, avoid electrical short circuits, and allow the transportation of charge carriers. At present, most research for AZIBs use a glass fiber (GF) separator, which is poor in mechanical properties, possessing large and uneven pores. To substitute GF, MOF-based materials have recently been progressively adopted as novel functional membranes. For instance, Yang and coworkers exploited the metal-organic framework Zn-BTC as an ionic sieve membrane for long-life aqueous zinc–iodine batteries (Figure 9a) [81]. As revealed by Raman spectroscopy, the Zn-BTC membrane can effectively suppress the shuttling of I_3_^−^ and regulate the electrolyte solvation structure with more aggregative ion association (Figure 9b). Consequently, the parasitic HER process was constrained, which beneficially conserved the smooth and dense Zn anode surface after cycling (Figure 9c). Compared with the GF separator, micro-infrared (IR) spectroscopies proved that the generation of ZnO and Zn(OH)_2_ containing a passivation layer was restrained with the assistance of the Zn-BTC membrane. Benefitting from these enhancements, symmetric Zn half cells in cooperation with the Zn-BTC membrane remarkably survived up to 3000 h with a stable potential profile in common 2 M ZnSO_4_ electrolyte. Moreover, the GF separator modified method is feasible as well. For example, a functionalized glass fiber separator with metal–organics UiO-66 frameworks was constructed by Song et al. (Figure 9d) [82]. They soaked the GF separator in UiO-66 synthetic solution to obtain MOFs in situ grown as novel separators (denoted as UiO-66-GF). The UiO-66 featuring a large specific surface area (990.3 m^2^ g^−1^) and porous structure equipped the UiO-66-GF membrane with a high transport ability for charge carriers. The electrical field distribution models based on GF and UiO-66-GF separators were analyzed by COMSOL finite element simulations. As illustrated in Figure 9e, the UiO-66-GF separator exhibited a more uniform electrical field than the GF separator, promoting the accomplishment of a uniform Zn plating/stripping. By measuring XRD patterns of the Zn anode before and after cycling, it was elucidated that Zn^2+^ deposition demonstrates a (002) crystal plane preferred orientation under the control of the UiO-66-GF membrane, which was conducive to inhibiting dendrites. Further analysis of DFT calculation dates explained the adsorption energies between H and Zn (002), (100), and (101) crystal planes are −1.731 eV, −1.954 eV, and −2.369 eV, respectively. A weaker adsorption of H by the (002) plane was favorable for HER suppression and outstanding corrosion resistance. Significantly, the Zn//UiO-66-GF//MnO_2_ cell presented a specific capacity of 230.8 mAh g^−1^ at 100 mA g^−1^, with excellent capacity retention up to 85% after 1000 cycles at 1.0 A g^−1^ (Figure 9f). Similarly, Maeboonruan et al. coated UiO-66 solution into a GF separator by dip coating (Figure 9g), effectively guiding uniform and dendrite-free zinc deposition as well [83]. Obviously, MOF-based materials have an exciting impact on the design of novel separators, which considerably improve the cycling stability of AZIBs via suppressing harmful dendritic growth and corrosion. However, almost all the separators of AZIBs suffer from poor tensile strength and Young’s modulus. It is promising to make progress toward the research direction of the MOF-based separators with good mechanical properties for AZIBs.

### 3.4. MOF-Based Materials in Electrolyte Engineering

**Figure 10:**
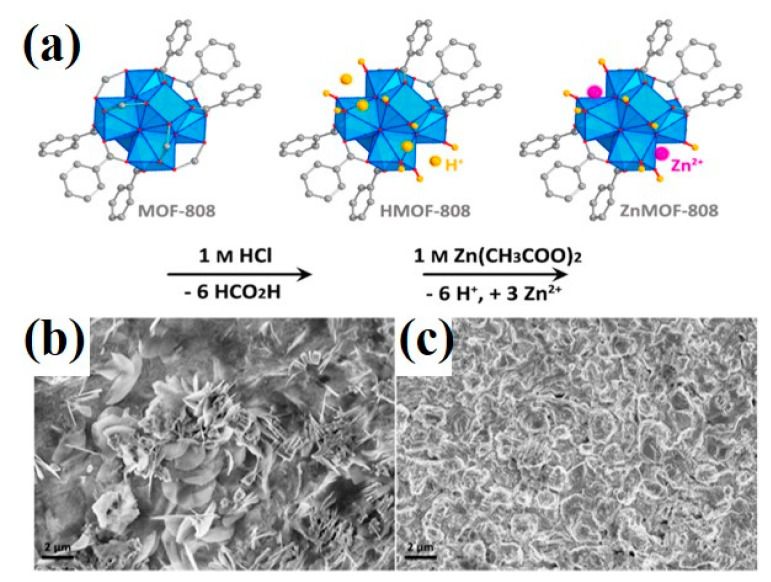
(**a**) Scheme for the post-synthetic of ZnMOF-808. SEM images of the Zn foils after plating/stripping cycles of (**b**) Zn|ZnSO4|Zn cell and (**c**) Zn|WZM|Zn cell [84]. Copyright 2019, Elsevier.

In the AZIBs, a narrow electrochemical stability window can be broadened in electrolyte engineering. For instance, Wang et al. originally reported a ZnMOF-808-based single-ion Zn^2+^ solid-state electrolyte (SSE), leading to dendrite-free rechargeable AZIBs (Figure 10a–c) [84]. The inherent conductivity of ZnMOF-808 was 3 × 10^−7^ S cm^−1^. However, after absorbing water, the water@ZnMOF-808 (denoted as WZM) SSE showed high conductivity up to 2.1 × 10^−4^ S cm^−1^, which was favorable for ion migration. The redox reaction mechanism in WZM SSE was identical to that in an aqueous ZnSO_4_ electrolyte, namely Zn^2+^ + 2e^−^ ⇌ Zn. Moreover, a high electrochemical window of 2.2 V and Young’s modulus of 5.3 GPa for WZM SSE was attained. Inspiringly, the VS_2_//WZM SSE//Zn battery delivered a reversible capacity of 125 mAh g^−1^ over 250 cycles at 0.2 A g^−1^ with 40% capacity retention at 2 A g^−1^ vs. 0.1 A g^−1^, further verifying the good properties of WZM SSE. Apparently, MOF-based materials exhibit a comparable redox activity in regulating Zn plating/striping behavior in electrolyte engineering, which is beneficial to high-performance AZIBs. However, there is little literature about employing MOF-based materials as electrolyte additives, which deserve to be further studied.

## 4. Summary and Perspectives

In conclusion, metal-organic frameworks (MOFs) are a class of porous materials composed of metal ions and organic linkers. They are attracting a great deal of attention in various fields due to their unique structures, tunable pore sizes, and large surface areas. In recent years, MOFs and their derivatives have also been studied in energy storage, especially in rechargeable aqueous zinc-ion batteries. All of the studies mentioned above have demonstrated that MOF-based materials show promising potential for use in Zn-ion batteries due to their ability to effectively store and release Zn^2+^ ions, prominently enhancing the capacity, stability, and overall performance of AZIBs whether in regard to cathode preparation or anode protection (Table 1).

However, in contrast to considerable reports on MOFs and their derivatives toward LIBs, the use of MOF-based materials in AZIBs is still in the initial research stage. Although MOF-based materials can improve the low capacity and poor stability of AZIBs, there are also several drawbacks that need to be addressed. More work is needed to fully understand the outlook of MOF-based materials in the field of AZIBs. The current major limitations and future prospects are elaborated as follows:

(1) Synthesis and cost. Currently, most MOF-based materials are synthesized in the complicated high-temperature and time-consuming conditions, which can make their large-scale production challenging and costly. Therefore, new efficient and cost-effective synthesis methods or synthesis optimizations are necessary, which help to overcome the current limitations of their production and then promote the practical application of AZIBs.

(2) Stability. MOF-based materials have a tendency to undergo pore collapse or size reduction during the operating process, which can reduce their surface area and negatively affect their performance in AZIBs. Moreover, many MOF-based materials are highly sensitive to water and can lose their structural integrity when exposed to a weakly acidic aqueous electrolyte. Hence, it is imperative to design MOF-based materials with improved structural stability and robustness.

(3) Conductivity. The majority of MOF-based materials deliver poor electric conductivity because of insulating organic ligands and the limited overlap between the p orbitals of organic linkers and the d orbitals of the metal ions [85,86]. In combination with other conductive materials such as rGO, it will make their widespread use cost prohibitive. Therefore, efforts are needed to design new MOF-based materials with improved properties.

(4) Energy density. Among MOF-based materials, PBAs have a high discharge platform but relatively low capacity. In the meantime, V-/Mn-/Zr-based MOFs possess relatively high capacity but low discharge voltage. Due to the energy density being decided by capacity and discharge voltage, it is commonly reported that AZIBs with MOF-based materials deliver low energy density, which significantly hinder their practical applications. Hence, enhancing the energy density of AZIBs is a vital research direction and worth further exploring.

Undoubtedly, the research on MOF-based materials in aqueous zinc-ion batteries is gradually maturing. Based on serving as cathode preparation and anode protection, MOF-based materials help AZIBs to overcome hostile challenges, making them more practical and efficient for future large-scale energy storage systems.

## Figures and Tables

**Table 1:** List of the electrochemical performances of MOF-based materials in AZIBs.

Samples	Roles	SC/R	RT/C/R	Ref.
ZnHCF	Cathode	65.4/0.06	75/100/0.3	[49]
Cu_3_(HHTP)_2_	Cathode	228/0.05	75/500/4	[50]
Mn(BTC)	Cathode	112/0.05	92/900/1	[51]
MIL-47	Cathode	332/0.1	58/70/1	[52]
Mn-H3BTC-4	Cathode	138/0.1	93.5/1000/3	[53]
CoMn-PBA HSs	Cathode	128.6/0.05	76.4/1000/1	[54]
CuMn-PBA DSNBs	Cathode	116.8/0.1	96.8/2000/1	[55]
PFC-8	Cathode	110/30	77.2/950/10	[56]
HCNS	Cathode	295.7/0.5	87/1500/1	[65]
α-Mn_2_O_3_	Cathode	225/0.05	53.3/1700/2	[69]
Cu-HHTP/MX	Cathode	260.1/0.1	92.9/1000/4	[42]
OH-rich MnHCF	Cathode	136.1/0.1	60.3/80/0.1	[73]
HVPO	Cathode	96.1/0.1	100/20,000/10	[74]
VN/N-CNFs	Cathode	297/100	100/30,000/50	[75]
ZIF-8	Anode	132/0.1	72/20,000/4	[76]
MDC	Anode	459/0.5	92/900/3	[77]
Zn@Zn-BTC	Anode	220/0.5	81.1/1000/3	[78]
Zn@UiO-66-(COOH)_2_	Anode	323/0.2	91/2400/1	[80]
Zn-BTC	Separator	201.1/0.16	99/100/0.16	[81]
UiO-66-GF	Separator	230.8/0.1	85/1000/1	[82]
ZnMOF-808	Electrolyte	125/0.2	89/250/0.2	[84]

SC: specific capacity (mAh g^−1^), R: rate (A g^−1^), RT: retention (%), C: cycles.

## Data Availability

No new data were created in this work. Data sharing is not applicable to this article.
